# Desmoid Tumors in Pregnant and Postpartum Women

**DOI:** 10.3390/cancers4010184

**Published:** 2012-02-21

**Authors:** William A. Robinson, Colette McMillan, Amy Kendall, Nathan Pearlman

**Affiliations:** 1 Department of Medicine, University of Colorado Denver, Denver, CO 80045, USA; 2 Department of Pharmacy, University of Colorado Denver, Denver, CO 80045, USA; E-Mails: colette.mcmillan@uch.edu (C.M.); 3 Department of the Tumor Registry, University of Colorado Denver, Denver, CO 80045, USA; E-Mails: amy.kendall@uch.edu (A.K.); 4 Department of Surgery, University of Colorado Denver, Denver, CO 80045, USA

**Keywords:** desmoid, pregnancy, post-partum, female

## Abstract

We report here a review of the current medical literature on pregnancy associated desmoids, including 10 cases of our own. The pertinent findings are that a large percentage of desmoids in females arise in and around pregnancy. Most occur in the abdominal muscles, particularly the right rectus abdominus, perhaps related to trauma from abdominal stretching and fetal movement. While these tumors may regress spontaneously after delivery most can be surgically resected with low recurrence rates even with R1 resections and this is clearly the treatment of choice. Subsequent pregnancies do not appear to result in recurrence in either FAP or non FAP patients. It is not clear from currently available data whether pregnancy associated desmoids are molecularly distinct from other desmoids.

## 1. Introduction

Pregnancy associated desmoids tumors have been a subject of interest since the first description by Macfarlene in 1832 [1]. He described a young postpartum woman who had a large fibrous abdominal wall tumor. This was also the first description of surgical resection of a pregnancy associated desmoid. Since then there have multiple single case reports and a few small series including one from our institutions [2]. There have been various definitions of pregnancy associated desmoid. For the purpose of this paper we have elected to define as pregnancy associated desmoids those that arose, or were discovered during pregnancy, grew during pregnancy or developed postpartum within three years after delivery. The reason(s) for the association between pregnancy and development of desmoids remain unclear. A number of possible explanation have been put forward, none very satisfactory. In a previous paper we described four women who all developed post-partum desmoids in the same location, namely the right rectus abdominus muscle. We speculated that this might have resulted from tearing or stretching of the abdominal wall musculature during fetal growth. It is well known that desmoids may be associated with trauma of different sorts. Against such a theory is the fact that in other case reports of pregnancy associated desmoids the tumor has developed in a remote location such as the larynx [3,4,5,6,7,8,9,10,11,12,13,14,15,16,17,18,19,20,21,22]. Others have attempted to link the hormonal and immune system changes that occur with pregnancy with desmoid development. The possible role of these factors is suggested by the description of spontaneous desmoid regression after delivery [23]. Further the possible role of estrogen is suggested by the occasional report of desmoids tumors responding to treatment with the anti-estrogen tamoxifen [24,25]. In contrast however is the finding that it is very uncommon to find estrogen receptors expressed on desmoids, even those that respond to tamoxifen. To date therefore the only thing clear in this unique association is that no clear explanation has been forthcoming. Here we have reviewed our own cases of pregnancy associated desmoids and the current literature in an attempt develops better understanding and future directions for research and management.

## 2. Methods

All cases with histologically proven desmoid tumors in the Tumor Registry of our institution from 1980 through August 2011 were initially identified. There were 56 total cases of which there were 24 males and 32 females. Among the females 10 fit our criteria for pregnancy associated desmoid: arising in pregnancy, growth of a previously identified desmoid during pregnancy or development in 36 months after parturition. We used this definition since these are slowly growing tumors and it is likely that they were present at the time of pregnancy. Thus in our series 18% of all patients seen here during this time period had a pregnancy associated desmoid. Perhaps more striking is the fact that of all females with desmoids in our registry 31% were pregnancy associated. Details of these patients are shown in Table 1. Four of these were included in a prior paper [2]. In the current series the patients ranged in age from 22 to 39 years with a mean age of 32 and a median age of 33 years. Nine of the 10 cases developed the tumor after completion of pregnancy at an average of 12 months postpartum. One was found in the last month of pregnancy. The most common site was the right rectus abdominus muscle in 6 cases; two were in the left rectus abdominus, one in the mesentery and one on the back in a patient with Gardner’s Syndrome. Surgical resection was the method of primary treatment in 10 patients. The tumors ranged in size from 2 to 16 cm with an average size of 4.5 cm. Two of these had microscopically positive margins, but none of the patients who had surgical resection; even with positive surgical margins have had local or systemic recurrence at a mean follow-up of 3.4 years. No patient received further therapy after surgery. One patient was treated with tamoxifen followed by gleevec prior to surgery with minimal benefit. Two patients subsequently had full term pregnancies without complications or desmoid recurrence. Two patients have active tumors not thought to be amenable to surgical resection and are currently being treated with tamoxifen and clinoril. Except for the patient with Gardner’s Syndrome no patient had a family history of desmoids tumors.

**Table 1:** Clinical characteristics of 10 patients with pregnancy associated desmoid tumors seen at the University of Colorado.

Patient	Age	FAP	Pre or post	Site	Treatment	Delivery	ER	Size (cm)	Outcome
1	39	No	Post 12 mon	Rt rectus	Surg	CS	NA	5	NED 5 years
2	39	No	Post 1 mon	Rt rectus	Surg	CS	NA	2	NED 5 years
3	33	No	Post 36 mon	Rt rectus	Surg	CS	NA	7.5	NED 2 years
4	32	No	Post 12 mon	Rt rectus	Surg	Vag	NA	16	NED 5 years
5	36	No	Post 13 mon	Rt rectus	Surg	CS	NA	2	NED 6 years
6	33	No	Post 4 mon	Left rectus	Surg	Vag	Neg	8	NED 2 years
7	26	Yes	Post 12 mon	Back	Tam/chemo	Vag	Neg	37	Alive with 2 years
8	33	No	Preg 40 wk	Left rectus	Tam/chemo	CS	Neg	8	Alive with 1 years
9	29	No	Post 6 mon	Mesentery	Surg	CS	NA	5	NED 3 years
10	22	No	Post 25 mon	Rt rectus	Surg	CS	NA	7	NED 3 years

Age range: 22–39 years; Mean: 32 years; mon: month(s), wk: weeks; Rt rectus: right rectus; Size range: 2–37 cm, Mean: 9.75 cm; NED: No evidence of disease. Alive = alive with tumor remaining.

## 3. Literature Review

We reviewed the available literature for reports of pregnancy associated desmoids and this data, minus our own cases, is detailed below in Table 2 [3,4,5,6,7,8,9,10,11,12,13,14,15,16,17,18,19,20,21,22]. We included only those reports in which there was sufficient data on the age of the patient, size of the tumor, location and method of treatment. We also found an abstract describing 15 cases, but this series has not been reported in a full manuscript and is described separately below. For the review we found 20 full length reports in the literature with a total of 24 cases that fit the criteria described and provided sufficient data for analysis. The patients ranged in age from 17 to 42 years with an average age of 27. Seven cases were associated with FAP mutations. The remaining were either non FAP or no mention was made of the genetic status. In 15 cases the desmoid arose during pregnancy, in five during the post-partum period and in four patients a pre-existing desmoid grew during pregnancy. In 14 of the 24 cases (58%) the desmoid involved the muscles of the abdominal wall. The mesentery was the next most common site and the remainder were in a wide variety of locations including the larynx in one case. Most were in the 5 to 10 cm size range, but one in case a tumor in the rectus abdominus grew to 192 cm at 39 weeks gestation. Most of these patients were treated with surgical resection Due to inadequate reporting we are unable to make definitive comments regarding outcome and recurrence in patients other than our own described above.

In addition to these cases Rocha *et al.* [23] have described, in abstract form, limited details of pregnancy associated desmoids seen at The Brigham and Women’s Hospital in Boston. They reviewed 207 total cases of desmoid tumors in their registry and identified 16 of these (8%) as having a pregnancy associated desmoid. Similar to our series, described above, most (75%) were extraabdominal, but the individual sites were not specified. None of these were FAP associated. All 16 patients had surgical resection and of these nine (56%) were R0 and 7(44%) were R1 resections. At a median follow-up of 39 months only two patients in this series had recurred, both after initial R1 resections. Both underwent successful re-excision. These authors concluded that pregnancy associated desmoids represent a less aggressive, subtype of desmoid tumors usually amenable to surgical resection.

**Table 2:** Pregnancy associated desmoid. A review of the literature.

Author(s) Ref.	Age of Patient	FAP	Number of Pregnancies	Delivery	Pre, During, or Post Pregnancy	Size	Location	Treatment	Outcome	ER
Ober *et al.* [3]	18	N/A	1	(unknown)	Pre	1–2 cm	Left popliteal fossa	Surg	NED 1 year	N/A
Khoo, S.K. [4]	25	No	2	Vag	During	8 × 5 × 3 cm	obturator	Surg	NED 6 months	
	35	N/A	2	CS	During 11 week gestation	(unknown)	Right obturator	No treatment	N/A	
Caldwell, E.H. [ 5]	26	N/A	4	(unknown)	Post	15 × 17 cm	Lower abdominal wall	No treatment	NED 5 years	N/A
Knightly *et al*. [6]	18	N/A	1	CS	Post	(unknown)	Right upper quadrant	Surg; XRT; chemo	(unknown)	
Harvey *et al*. [7]	23	Yes	1	(unknown)	During 3rd trimester	N/A	Mesentry	Surg	(unknown)	
	24	Yes	1	(unknown)	Post	12 × 10 × 5 cm	Mesentry	Surg	NED 3 years	
	34	Yes	1	(unknown)	Post	(unknown)	Terminal ileus	Surg	(unknown)	
Camiel & Solish [8]	22	No	2	(unknown)	During 7th month	14 × 10 cm	Right, upper anterior abdominal wall	Surg	N/A	
Ezra *et al*. [9]	35	No	1	Vag	Pre	15 × 13 cm	Abdominal wall	Surg	NED 2 years	N/A
Sportiello & Hoogerland [10]	40	N/A	1	CS	During 3rd trimester	10 × 8 × 6 cm	Pelvic mass	Surg; XRT; chemo	NED at 27 months	N/A
Allen & Novotny [ 11]	19	No	1	(unknown)	Pre	3 × 3 cm	Right labium	Surg; XRT	NED at 12 months	
Kunieda *et al*. [12]	27	N/A	1	(unknown)	During 8th month	9 × 7.5 cm	Chest wall, xphoid	Surg	NED	Neg
Way & Culham [ 13]	28	N/A	3	Vag	Post	3 × 4 × 2.5 cm	Left rectus abdominal muscle	Surg	NED	
Way & Culham [ 13]	28	N/A	1	(unknown)	Post	1.3 × 1.2 cm	Left upper rectus muscle just below the costal margin	Surg	NED	Neg
Gherman *et al.* [14]	25	N/A	1	Vag	During 20 weeks gestation	2.3 × 1.2 × 1.6 cm	Larynx	Surg	(unknown)	
de Cian *et al*. [15]	42	N/A	2	CS	During 12 weeks gestation	8 × 5 cm	Right rectus abdominus c-section scar	Surg	NED 15 months	NEG
Firoozmand & Prager [16]	27	Yes	1	(unknown)	During 23 weeks gestation	17 × 14 × 10 cm	J pouch	Surg	N/A	N/A
Mulik *et al.* [17]	35	Yes	5	Vag; CS	During 18 weeks gestation	5 × 4 cm4 × 4 cm	Rectus sheath	Surg	No follow up	
Molelekwa, V. [18]	31	N/A	2	CS	During 20 weeks gestation	2.7 × 4.5 cm	Left lower quadrant abdominal mass	Surg	N/A	
Durkin *et al.* [19]	29	N/A	1	Vag	During 1st trimester	3.5 × 7.2 cm	Left rectus abdominis muscle	Surg; chemo	NED 2.5 years	
Sun *et al.* [20]	28	N/A	1	CS	Post	12.2 × 11.5 × 8.5 cm	Mesentery abdomen	Surg	NED 12 months	
Viriyaroj *et al.* [21]	17	N/A	1	(unknown)	During 5th month	28 × 21 × 18 cm	Lower abdominal wall	Surg	NED 8 months	
Le Roc'h *et al.* [22]	18	N/A	1	Vag	Pre	192 cm^3^	Rectus muscle of the abdomen	No treatment	N/A	N/A

## 4. Discussion

We review here 50 cases of pregnancy associated desmoid tumors including 10 cases from our own institution. These appear to be a unique form of desmoids comprising 8 to 18% of all desmoid tumors in cancer registries. They are usually sporadic but may be associated with the FAP syndrome. If we exclude patients with FAP there does not appear to be any common features that mark women at risk for this problem. The patients in our series were perhaps older (mean age 32 years) than pregnant women without desmoids. The age(s) were not given for the patients in the series reported by Rocha *et al.* [23]. Most, patients, both in our own series and our review had a prior pregnancy often with a caesarian section. While desmoids have been reported to arise in caesarian section scars this appears to be uncommon. The most common site is in the abdominal musculature particularly the right rectus abdominus muscle. Since these tumors are known to arise in areas of trauma we have speculated that this results from stretching of the abdominal muscles and fascia during gestation. This suggestion of causation does not explain however the development of desmoids in other areas during and after pregnancy indicating that other factors are also involved. The most likely factors are the hormonal and immunologic changes that occur during pregnancy.

The fact that some desmoids have been reported to regress without treatment after termination of pregnancy indicates that such factors may be in play. The most important of these are probably hormonal. This suggestion is strengthened by reports of spontaneous regression of pregnancy associated desmoids after delivery and without treatment. Further are many reports that some desmoids, both in pregnant and non-pregnant patients, respond to treatment with the anti-estrogen tamoxifen or the related drug toremifene [24]. This is not a simple estrogen related phenomenon however. Very few desmoids have been reported to express significant estrogen receptors. This may be due to inadequate methods of investigation in the past. These receptors are almost always present if carefully searched for, although their significance is unknown. The effects of tamoxifen are considerably more broad and complex than blocking estrogen uptake [25]. The doses used for desmoids are usually much higher (200 mg/day) [25] than typically used in the treatment of breast cancer (20 mg/day) and usually combined with a non-steroidal anti-inflammatory drug such as sulindac. The response rate to tamoxifen therapy in all desmoids is however in the range of 50% [24] and understanding the mechanisms involved is likely to lead to better understanding of development and new therapies. Finally regression of desmoids has been reported to occur in women as they go through menopause.

In contrast prior pregnancy has been reported to ameliorate the course of intra-abdominal desmoids in patients with FAP [26]. These authors compared the growth and progression of FAP related desmoids in 22 never pregnant patients with 25 similar patients who had been pregnant at least once. The patients who had been pregnant had smaller tumors, less surgery and a more benign course overall. These authors go as to far as to suggest that consideration should be given to possible therapy with hormone combinations of progesterone, prolactin and estrogen. It should be noted that these findings were reported only for FAP related desmoids and whether they are applicable to sporadic pregnancy associated desmoids is not clear.

In order to better understand the mechanisms involved in the development of desmoids and develop new therapies a number of studies have examined molecular pathways, though not specifically in pregnancy associated desmoids. Heinrich *et al*. [27] examined tumor specimens from 19 patients looking for possible imatinib targets. No mutations were found in KIT, PDGFRA or PDGFRB, but 16 of the 19 had mutations involving the WNT pathway (APC or CTNNB1). These findings provide a link to the development of spontaneous desmoids and those occurring in FAP patients as the same pathways are involved. These findings were confirmed by Salas *et al.* in a larger study of 194 patients [28] using comparative genomic and DNA sequencing. This study included 13 women of child bearing age, during or shortly following pregnancy. Of these detailed molecular data is reported for only three. Two of the three patients did not have mutations of CTNNB1, but did have loss of parts of 5q including the area encoding APC. Together these findings provide a link to the development of spontaneous desmoids and those occurring in FAP patients as the same pathways are involved. Although longer term studies are needed it would appear that those patients without mutations of CTNNB1, such as pregnancy associated desmoids, have a more favorable prognosis for unclear reasons. These same authors [29] have recently reported on outcome and prognostic factors in 426 patients with desmoid tumors. Of these 32 (7.5%) had abdominal wall pregnancy associated desmoids with a 10 year progression survival of 62%, the highest of any subgroup in this large series.

One of the questions frequently asked by patients with pregnancy associated desmoids is what the risk of recurrence with subsequent pregnancies is. We could not find any non FAP cases in which this occurred. In our own series two patients had subsequent pregnancies, Rocha’s series had one [23] and Way *et al*. [13] reported two further patients with no recurrence with a subsequent pregnancy. Of interest in this regard is a patient reported by Caldwell [5] who described a 26 year old woman who a large, unresectable desmoid during pregnancy which regressed without therapy even during a subsequent pregnancy.

## 5. Conclusions

Desmoid tumors in females are often associated with pregnancy or occur post-partum. The reasons behind this association are unclear. The most common sites are in the abdominal muscles, but they have been reported in multiple other areas of the body. Surgical resection is the treatment of choice and is usually curative even when surgical margins are involved. Subsequent pregnancies do not appear to be contraindicated.
